# Alanine transferase levels (ALT) and triglyceride-glucose index are risk factors for type 2 diabetes mellitus in obese patients

**DOI:** 10.1007/s00592-023-02209-6

**Published:** 2023-12-06

**Authors:** Franco Folli, Antonio E. Pontiroli, Ahmed S. Zakaria, Lucia Centofanti, Elena Tagliabue, Lucia La Sala

**Affiliations:** 1 Dipartimento di Scienze della Salute, Università degli Studi di Milano, Milan, Italy; 2 Ospedale San Paolo, Milan, Italy; 3 Laboratory of Cardiovascular and Dysmetabolic Diseases, IRCCS MultiMedica, Milan, Italy

**Keywords:** Liver, Type 2 diabetes, Triglyceride-glucose index, Metabolic syndrome

## Abstract

**Aims:**

The role of liver steatosis and increased liver enzymes (ALT) in increasing incident type 2 diabetes mellitus (T2DM) is debated, because of their differential effects on different ethnicities and populations. The aim of this study was to evaluate the role of elevated ALT in the development of T2DM in non-diabetic obese subjects receiving routine medical treatment.

**Methods:**

A total of 1005 subjects [296 men and 709 women, aged 45.7 ± 13.12 years, body mass index (BMI) 39.5 ± 4.86 kg/m^2^] were followed for a mean period of 14.3 ± 4.44 years. Subjects were evaluated for several metabolic variables, including the triglyceride-glucose index and the presence of metabolic syndrome (IDF 2005 definition), and were subdivided into ALT quartiles.

**Results:**

T2DM developed in 136 subjects, and the difference was significant between the first and the fourth ALT quartile (*p* = 0.048). Both at univariate analysis and at stepwise regression, ALT quartiles were associated with incident T2DM. Traditional risk factors for T2DM coexisted, with a somehow greater predictive value, such as triglyceride-glucose index, age, arterial hypertension, LDL-cholesterol, and metabolic syndrome.

**Conclusions:**

These data suggest an association between elevated ALT levels and the risk of incident T2DM in obesity.

## Introduction

Several cross-sectional and prospective studies have shown that deterioration of glucose tolerance and incidence of type 2 diabetes mellitus (T2DM) in the general population are associated with elevated liver enzymes, even though with some discrepancies among different ethnic groups [1–7]; ALT was the main enzyme involved in the majority of studies, while in others the enzyme was **γ**GT (**γ**-glutamil transferase) [2–7]. Obesity is often accompanied by elevated liver enzymes [5, 6, 8], and bariatric surgery (BS) is associated with decreases of body weight, ALT and AST levels, and with a reduced incidence of T2DM [8]. However, in obese subjects only cross-sectional studies have evaluated the association of T2DM with elevated liver enzymes [5, 6]. To better understand the possible role of liver enzymes in incident diabetes, in this study we retrospectively analyzed two cohorts of obese subjects that had been already evaluated for co-morbidities and mortality after routine medical treatment or BS [8, 9].

## Materials and methods

This study is a second analysis of two studies aimed at evaluation of prevention of co-morbidities and of mortality in subjects undergoing BS versus control subjects receiving routine medical treatment, and details of the studies have already been published [8, 9]. It is important to remember that the National Health System (NHS) covers more than 95% of all hospital admissions, medical and surgical procedures and medical expenses of citizens [10] (Italian Survey 2012). The Regional Lumbardy Administrative Database contains since 1988 all pertinent data of all citizens, and in particular, the Lumbardy database collects several information, including (1) an archive of residents who receive NHS assistance, reporting demographic and administrative data; (2) a database on diagnosis at discharge from public or private hospitals of the region; (3) a database on outpatient drug prescriptions reimbursable by the NHS; and (4) a database on outpatient visits, including visits in specialist ambulatory care and diagnostic laboratories accredited by the NHS. For each patient, these databases are linked through a single identification code. The Italian National Health System keeps record of all acute and chronic diseases (diabetes mellitus, liver and cardiovascular diseases, selected thyroid, renal, and lung diseases, among others). This system yields the right to exemption from medical charges (exemptions), that means life-long free prescriptions and examinations for any disease. Therefore, together with hospital admissions, exemptions were considered a proxy of development of chronic diseases. This procedure has previously been employed and validated [8, 11, 12].

For this study, we considered only obese subjects without diabetes at baseline receiving routine medical treatment; subjects undergoing BS were excluded from the study, because BS can prevent incidence of T2DM [8]. Therefore, we analyzed a total of 1005 subjects (296 men and 709 women, aged 45.7 ± 13.12 years) which were followed for a mean period of 14.3 ± 4.44 years. Clinical, and laboratory characteristics of subjects under study are shown in Table 1, where subjects have been subdivided according to ALT quartiles. Diagnosis of incident diabetes was based on exemption together with date of exemption, and therefore only clinical diabetes was considered. The presence of the triglyceride-glucose index (TYG) was included [13], and metabolic syndrome was estimated according to the 2005 IDF definition [14].

**Table 1:** Clinical and laboratory data of non-diabetic obese subjects recruited as controls of bariatric surgery, divided according to quartiles of liver enzymes (ALT)

	1^st^ quartile *N* = 265	2^nd^ quartile *N* = 259	3^rd^ quartile *N* = 246	4^th^ quartile *N* = 235	*p*
Age (years)	45.5 ± 13.44	47.7 ± 12.78	46.1 ± 13.58	43.2 ± 12.01	0.0014
Sex (males/females)	27/238	48/213	82/148	139/110	0.0001
BMI (kg/m^2^)	39.5 ± 4.81	39.4 ± 4.71	39.7 ± 4.97	39.1 ± 5.17	0.5999
Waist circumference (cm)	108.8 ± 9.04	113.7 ± 11.14	115.1 ± 12.01	115.6 ± 11.12	0.0001
Blood glucose (mg/dl)	94.4 ± 11.31	96.5 ± 12.26	95.6 ± 12.32	98.1 ± 12.13	0.0056
Glucose triglyceride index*	8.6 ± 0.53	8.7 ± 0.44	8.7 ± 0.51	8.9 ± 0.55	0.0001
Glucose tolerance (NGT/IFG)	189/74	176/84	163/83	137/93	0.0360
Hypertension (yes/no)	85/180	90/171	80/150	69/180	0.3051
Therapy for hypertension (yes/no)	46/219	52/209	53/196	27/203	0.0334
Systolic BP (mmHg)	139.2 ± 21.02	138.0 ± 18.41	140.9 ± 20.87	140.8 ± 18.92	0.6231
Diastolic BP (mmHg)	84.9 ± 10.48	84.5 ± 10.04	86.9 ± 10.01	86.5 ± 11.45	0.2192
Total-cholesterol (mg/dl)	208.8 ± 39.90	217.4 ± 39.08	219.1 ± 49.44	216.6 ± 45.91	0.3943
HDL-cholesterol (mg/dl)	54.2 ± 13.36	52.1 ± 12.43	49.2 ± 12.09	47.4 ± 16.72	0.0001
LDL-cholesterol (mg/dl)	132.8 ± 40.27	142.2 ± 40.08	143.2 ± 39.01	137.9 ± 39.60	0.3560
Triglycerides (mg/dl)	130.0 ± 108.81	139.0 ± 61.15	145.1 ± 78.35	172.7 ± 114.14	0.0001
AST (U/L)	17.0 ± 4.69	21.4 ± 5.94	25.3 ± 7.19	38.5 ± 17.73	0.0001
ALT (U/L)	15.4 ± 2.97	23.9 ± 2.52	33.8 ± 3.49	64.3 ± 22.71	0.0001
**γ**GT (U/L)	20.1 ± 6.60	25.04 ± 9.27	44.1 ± 12.21	48.6 ± 23.95	0.0002
Metabolic syndrome (yes/no)	187/78	200/61	186/44	205/44	0.007
EGFR (mL/min/1,73 mq)	87.7 ± 20.26	88.6 ± 23.36	88.5 ± 20.61	90.1 ± 21.71	0.6880

Mean ± SD or absolute frequency is reported

^*^Ln (fasting TG (mg/dL) × FBG (mg/dL)/2)

## Statistical analysis

Data of subjects in study were analyzed first as being part of ALT quartiles. One way analysis of variance (ANOVA) was employed to assess significant differences between data of subjects in the different ALT quartiles. Chi-square analysis was employed to assess distribution of subjects within each ALT quartile developing diabetes. Then, a univariate regression analysis was performed to assess association between each independent variable (all variables listed in Table 1) and incidence of diabetes. Finally, a stepwise regression was used to assess the risk of diabetes connected with independent variables statistically significant at univariate analysis. Various models of stepwise regression were used, with incident diabetes as the dependent variable (Table 2); age, sex, blood glucose, TYG index, glucose tolerance, arterial hypertension, LDL-cholesterol, ALT quartile, AST quartile, and metabolic syndrome were alternatively introduced as independent variables; triglycerides were generally omitted from models because of collinearity with age, TYG index, AST, ALT, and metabolic syndrome. *p* levels < 0.05 were considered statistically significant. All statistical analyses were performed through Stata, version 17.0, for MacIntosh.

**Table 2:** Risk of type 2 diabetes mellitus according to alanine transaminase (ALT) quartile

	Quartile of serum ALT levels*				*p* value for trend
	1^st^ quartile *N* = 265	2^nd^ quartile *N* = 261	3^rd^ quartile *N* = 230	4^th^ quartile *N* = 249	
N of events	27	30	39	40	
Crude HR	1.00	1.49	1.70	1.61	0.068
95% CI	Reference	− 0.045–0.072	0.001–0.128	0.000–0.012	
*p* value versus 1^st^ quartile	Reference	0.6301	0.0272	0.0480	

^*^1^st^ quartile = 5–19 U/L; 2^nd^ quartile = 20–28 U/L; 3^rd^ quartile = 29–40 U/L;4^th^ quartile = 41–178 U/L

## Results

Table 1 shows that with increase of ALT quartiles, waist circumference also increased, as well as blood glucose and the triglyceride-glucose index, LDL-cholesterol, triglycerides, ALT, and AST, ALT, and frequency of metabolic syndrome. In contrast, age and sex ratio were different in the different ALT quartiles, and HDL-cholesterol decreased; mean values of blood glucose were within the normal range of values. At chi-square analysis, incidence of diabetes progressively increased with greater ALT quartiles (27/265, 30/261, 39/230, and 40/249, respectively, *p* = 0.068), although not significantly; when only 1^st^ and 3^rd^ ALT or 1^st^ and 4^th^ quartiles were compared, the difference was statistically significant (*p* = 0.0272 and *p* = 0.048, respectively, Table 2). The low statistical significance is likely due to the small sample size; in fact, when the whole sample was doubled in a simulation analysis, the overall analysis yielded a *p* value = 0.0026. At univariate regression analysis, age, waist circumference, blood glucose, TYG index and glucose tolerance, arterial hypertension, systolic BP, diastolic BP, LDL-cholesterol, triglycerides, metabolic syndrome, ALT and ALT quartile, but not AST or **γ**GT, were all positively correlated with incident diabetes, while female sex, HDL-cholesterol and EGFR were negatively correlated with incident diabetes. At stepwise regression (Table 3), in Model 1, risk factors for incident diabetes were age, TYG index, LDL-cholesterol, ALT quartiles; when ALT quartiles are substituted by AST quartiles, only age (*p* = 0.043), TYG index (*p* = 0.001), and LDL cholesterol (*p* = 0.040) were statistically significant risk factors; # when TYG index was substituted by Blood Glucose, only Blood Glucose (*p* = 0.001) and LDL cholesterol (*p* = 0.016) were statistically significant risk factors. In Model 2, risk factors for incident diabetes were TYG index, LDL-cholesterol, and ALT quartile; when ALT quartiles are substituted by AST quartiles, only age (*p* = 0.009), TYG index (*p* = 0.001), and LDL cholesterol (*p* = 0.036) were statistically significant risk factors. In Model 3, age, ALT quartile, and metabolic syndrome were risk factors for incident diabetes; when ALT quartiles are substituted by AST quartiles, only age (*p* = 0.002), and Metabolic Syndrome (*p* = 0.001) were statistically significant risk factors. In Model 4, glucose tolerance, L-cholesterol, and ALT quartiles were risk factors for incident diabetes; when ALT quartiles are substituted by AST quartiles, only glucose tolerance (*p* = 0.001), and LDL cholesterol (*p* = 0.022) were statistically significant risk.

**Table 3:** Univariate regression analysis and stepwise regression of risk factors for type 2 diabetes mellitus in a population of obese subjects receiving routine medical treatment

Univariate regression analysis			Stepwise regression			
			Model 1*#	Model 2**	Model 3***	Model 4****
	*r*	*p*	*p*	*p*	*p*	
Age	0.1204	0.001	0.035	0.109	0.001	0.110
Female sex	− 0.0647	0.012				
Waist	0.1635	0.001				
Blood glucose	0.2918	0.001				
TYG index	0.1604	0.001	0.001	0.001		
Glucose tolerance (NGT/IFG)	0.1775	0.001				0.001
Hypertension	0.0701	0.007	0.063			
Therapy for hypertension (yes/no)	0.0519	0.044				
Systolic BP	0.0984	0.009				
Diastolic BP	0.0793	0.035				
HDL-Chol	− 0.0615	0.046				
LDL-Chol	0.0793	0.005	0.037	0.055		0.017
Triglycerides	0.1072	0.001				
Metabolic syndrome	0.0863	0.001			0.002	
EGFR	− 0.0796	0.012				
ALT	0.0625	0.047				
AST	0.122	0.122				
yGT	0.1559	0.167				
ALT quartile	0.0757	0.016	0.044	0.037	0.019	0.044
AST quartile	0.0627	0.046				

Triglycerides were generally omitted from models because of collinearity with age, TYG index, AST, ALT, and Metabolic Syndrome

*Model 1* Age, TYG index, hypertension, LDL cholesterol, ALT quartiles

(*When ALT quartiles are substituted by AST quartiles, only age (*p* = 0.043), TYG index (*p* = 0.001), and LDL cholesterol (*p* = 0.040) were statistically significant risk factors; # when TYG index was substituted by Blood Glucose, only Blood Glucose (*p* = 0.001) and LDL cholesterol (*p* = 0.016) were statistically significant risk factors)

*Model 2* Age, TYG index, LDL cholesterol, ALT quartiles

(**When ALT quartiles are substituted by AST quartiles, only age (*p* = 0.009), TYG index (*p* = 0.001), and LDL cholesterol (*p* = 0.036) were statistically significant risk factors)

*Model 3* Age, Metabolic Syndrome, ALT quartiles

(***When ALT quartiles are substituted by AST quartiles, only age (*p* = 0.002), and Metabolic Syndrome (*p* = 0.001) were statistically significant risk factors)

*Model 4* Age, glucose tolerance, LDL cholesterol, ALT quartiles

(****When ALT quartiles are substituted by AST quartiles, only glucose tolerance (*p* = 0.001), and LDL cholesterol (*p* = 0.022) were statistically significant risk)

## Discussion

In this study, markers of glucose metabolism (fasting blood glucose, TYG index, glucose intolerance and metabolic syndrome) and liver enzymes, mainly ALT, were predictors of incident diabetes in non-diabetic obese subjects receiving routine medical treatment over a mean follow-up of about 14 years. Raised liver enzymes, ALT and AST could be signs of MAFLD (NAFLD) [15] [16–19], and liver steatosis is a component of metabolic syndrome X/insulin resistance syndrome [18–22]. Therefore, it is not surprising that increased ALT levels were associated with incident diabetes in obese subjects receiving routine medical treatment. This association holds even at stepwise regression, albeit other traditional risk factors for diabetes coexist, with a somehow greater predictive value, such as TYG index, glucose tolerance, age, arterial hypertension, LDL-cholesterol, and metabolic syndrome, while blood glucose abolished the predictive role of ALT; of note, the fact that blood glucose levels were within the normal range of values, and that TYG index and glucose tolerance had a very strong predictive role. Of interest the fact that AST had no significant role, as already reported in other studies [3]; in contrast, cross-sectional studies in obese subjects [5, 6] reported a different role of ALT and **γ**GT, probably linked to the different size of the studies and to the different ethnic groups considered. These data suggest an association between glucose metabolism and elevated ALT levels and the risk of T2DM in obesity in obese non-diabetic subjects receiving routine medical treatment. We should also recall that decrease of liver enzymes after bariatric surgery is not universal, and that subjects not showing decrease of liver enzymes after BS are affected by a more severe insulin resistance than other subjects [23], and therefore are likely to be at risk of future incidence of T2DM. Our data add to cross-sectional [5, 6] and prospective [1–4, 7] studies showing a predictive role of liver enzymes in incident diabetes; however, these data probably represent a particular aspect of morbid obesity, and cannot be generalized; for instance, sex distribution of our study is different from studies in the general population, as it is for the cross-sectional study mentioned above [6]. It is likely that in different populations [1, 5, 6], and in different subjects, like gestational diabetes [24], other factors are of greater importance than liver enzymes.

## Abbreviations

ALT: Alanine transaminase
T2DM: Type 2 diabetes mellitus
BMI: Body mass index
AST: Aspartate transaminase
BS: Bariatric surgery
TYG: Triglyceride-glucose index
LDL: Low-density lipoprotein
EGFR: Estimated glomerular filtration rate
HDL: High-density lipoprotein
BP: Blood pressure
MAFLD: Metabolic dysfunction-associated fatty liver disease
